# Construction of HGF-Displaying Yeast by Cell Surface Engineering

**DOI:** 10.3390/microorganisms10071373

**Published:** 2022-07-07

**Authors:** Seiji Shibasaki, Yuki Nakatani, Kazuaki Taketani, Miki Karasaki, Kiyoshi Matsui, Mitsuyoshi Ueda, Tsuyoshi Iwasaki

**Affiliations:** 1 Laboratory of Natural Science, Faculty of Economics, Toyo University, Hakusan, Bunkyo-ku, Tokyo 112-8606, Japan; 2 Department of Diabetes, Endocrinology and Clinical Immunology, School of Medicine, Hyogo Medical University, Mukogawa-cho, Nishinomiya 663-8501, Japan; karasaki.1123@gmail.com (M.K.); k-matsui@hyo-med.ac.jp (K.M.); t-iwasaki@keiseikai.or.jp (T.I.); 3 School of Pharmacy, Hyogo University of Health Sciences, 1-3-6 Minatojima, Chuo-ku, Kobe 650-8530, Japan; ph08115@std.huhs.ac.jp (Y.N.); ph07082@std.huhs.ac.jp (K.T.); 4 Division of Applied Life Sciences, Graduate School of Agriculture, Kyoto University, Kitashirakawa-oiwakecho, Sakyo-ku, Kyoto 606-8502, Japan; ueda.mitsuyoshi.7w@kyoto-u.ac.jp

**Keywords:** cell surface, hepatocyte growth factor-displaying, yeast cells, oral administration, graft versus host disease

## Abstract

Hepatocyte growth factor (HGF) has been investigated as a regulator for immune reactions caused by transplantation and autoimmune diseases and other biological functions. Previous studies demonstrated that cDNA-encoding HGF administration could inhibit acute graft-versus-host disease (GVHD) after treatment via hematopoietic stem cell transplantation. This study aimed to show the preparation of HGF protein on yeast cell surfaces to develop a tool for the oral administration of HGF to a GVHD mouse model. In this study, full-length HGF and the heavy chain of HGF were genetically fused with α-agglutinin and were successfully displayed on the yeast cell surface. This study suggested that yeast cell surface display engineering could provide a novel administration route for HGF.

## 1. Introduction

Hepatocyte growth factor (HGF) is homologous to plasminogen, is secreted as the inactive form, and is activated by proteolytic processing [1]. In addition, HGF has a physiological function in hepatocytes and other cells. These functions include mitogenesis, migration, anti-apoptosis, and angiogenesis and play an important role in the regeneration and protection of tissues and organs [2,3,4]. Currently, HGF is important in clinical applications for acute organ diseases such as myocardial infarction, cerebral infarction, fulminant hepatitis, and acute renal failure [5,6,7]. The growth factor is therapeutic for chronic diseases such as pulmonary fibrosis, chronic renal failure, liver cirrhosis, cardiomyopathy, and arteriosclerosis obliterans [8,9,10].

In addition, graft-versus-host disease (GVHD) could be inhibited by HGF [11,12]. GVHD is caused by bone marrow transplantation or blood transfusion to treat leukemia, lymphomas, bone marrow failure syndrome, and immunodeficiency disorders [13]. Acute GVDH following an allogeneic hematopoietic stem cell transplant (HSCT) is an immune-triggered process, leading to a severe immune disorder and organ dysfunction caused by donor T cells [14]. However, donor cells also attack residual leukemic cells and host immune cells after HSCT, inhibiting leukemic relapse and graft rejection after HSCT. Therefore, to perform HSCT successfully, continuous donor T cell activation should be maintained to inhibit leukemic relapse, graft rejection, and organ dysfunction. Using a murine model of acute GVHD, Kuroiwa et al., demonstrated that the transfection of the human HGF cDNA into skeletal muscle inhibited apoptosis of intestinal epithelial cells and donor T-cell infiltration into the liver, thereby ameliorating the enteropathy and liver injury caused by acute GVHD [15]. The molecular structure of HGF is a heterodimer consisting of a heavy chain (α-chain; 69 kDa) and a light chain (β-chain; 35 kDa) [16]. By binding to tyrosine receptor c-Met, full-length HGF (84 kDa) plays a role in cell proliferation, migration enhancement, morphogenesis, and anti-apoptosis [2,17]. Conversely, the heavy chain of HGF, known as NK4, can bind to c-Met and function as an antagonist to HGF [18,19]. Therefore, the administration of full-length or heavy-chain HGF would aid in examining whether GVHD can be regulated by using an appropriate method.

In recent studies, a cell surface display system using microorganism cells [20] has been developed as a biotechnological tool to conveniently produce a foreign protein on the cell wall. Various host cells have been investigated for this cell surface display method, including *Escherichia coli*, *Lactobacillus casei*, *Bacillus subtilis* and *Saccharomyces cerevisiae* [21]. Among various microbial cells in a molecular display system, the budding yeast *S. cerevisiae* is the most suitable host for displaying a eukaryotic protein. Anchoring proteins for displaying a protein on the microbial cell surface have also been examined. For example, α-agglutinin, Aga1/Aga2, and Flo1 proteins were proven to function well to display a foreign protein on the *S. cerevisiae* cell surface [21,22]. 

Antigenic proteins have been investigated in order to produce oral vaccines as medicinal applications of cell surface display engineering. For example, antigenic proteins derived from *Candida albicans* [23,24], Influenza virus [25], or human papillomavirus [26] were displayed on cell surfaces using genetic engineering. Furthermore, displayed proteins could function as oral vaccines, enhancing immunological responses against target pathogens. In that regard, the oral administration of the HGF protein by the molecular display system might control GVHD [15].

In this study, we showed that the HGF construct could be displayed on the surface of *S. cerevisiae*. To anchor the HGF protein on the yeast cell surface, α-agglutinin was selected for the stable display based on a previous study [21,27]. Furthermore, the appropriate cultivation periods of yeast cells so as to produce the HGF protein on their surface were also examined. 

## 2. Materials and Methods

### 2.1. Strain and Media

The *E. coli* strain DH5α [F^—^, Φ80d*lacZ*ΔM15, Δ(*lacZYA-argF*)U169, *deoR, recA*1, *endA*1, *hsdR*17(r_K_^—^, m_K_^+^), *phoA*, *supE*44, λ^—^, *thi*-1, *gyrA*96, *relA*1] [28] was used as a host for the manipulation of recombinant DNA. The *E. coli* strain was grown in Luria–Bertani medium [1% (*w*/*v*) tryptone, 0.5% (*w*/*v*) yeast extract, 0.5% (*w*/*v*) NaCl, and 0.1% (*w*/*v*) glucose]. The *S. cerevisiae strain* BY4741 (*MATa his3-1 leu2 met15 ura3*) was used for the cell surface display of antigenic proteins. Yeast extract peptone dextrose medium [1% (*w*/*v*) yeast extract, 2% (*w*/*v*) peptone, and 2% (*w*/*v*) glucose] was used for the transformation of yeast cells. Yeast cells that carried a plasmid were grown in synthetic drop-out medium [2% (*w*/*v*) glucose, 0.67% (*w*/*v*) yeast nitrogen base without amino acids, 1% (*w*/*v*) casamino acids, and supplemented with appropriate amino acids]. The cell density was measured at 600 nm.

### 2.2. Plasmid Construction and Transformation of Yeast Cell

The plasmid pDHGF-FL used to display human HGF on the surface of *S. cerevisiae* cells was constructed by amplifying the full-length HGF-encoding sequence by polymerase chain reaction (PCR) using the following primers: Ffu, 5′-GTTTCTGCCAGATCTATGTGGGTGACCAAACTCCTGCCA-3′ and Rfu, 5′-AGATCCACCCTCGAGTGACTGTGGTACCTTATATGTTAA-3′ and human HGF cDNA [11]. The fragment of the gene encoding the full-length HGF was inserted into BglII/XhoI-digested pULD1 [29] using the In-Fusion HD Cloning kit (Clontech, Mountain View, CA, USA). Furthermore, pDHGF-HC, used for displaying the HGF heavy chain on the surface of *S. cerevisiae* cells, was constructed following the procedure used for pDHGF-FL. For the amplification of the heavy chain of HGF, the following primers were used: Ffu, 5′-GTTTCTGCCAGATCTCAAAGGAAAGGAAGAAATACAATT-3′ and Rfu, 5′-GAGTCCACCCTCGAGTCGCAATTGTTTCGTTTTGGCACA-3′. Thus, the full-length HGF-encoding sequence and the heavy chain of the HGF-encoding sequence were fused to the 5′ end of the cell-wall anchoring protein (α-agglutinin-encoding sequence) in these plasmids. The constructed plasmids were introduced in *E. coli* DH5α for propagation. Next, plasmids were introduced into *S. cerevisiae* BY4741 using the lithium acetate method [30] for the protein surface display. The nucleotide sequence of the constructed plasmids was confirmed using an ABI PRISM 3130 Genetic Analyzer (Applied Biosystems, Foster City, CA, USA).

### 2.3. Immunostaining

Yeast cells were collected by centrifugation at 6000× *g* for 5 min, washed with phosphate-buffered saline (PBS; 50 mM phosphate, 150 mM NaCl, pH 7.4), and adjusted to 3.2 × 10^8^ cells mL^−^^1^ with PBS. Next, 200 μL of the cell suspension was centrifuged at 6000× *g* for 5 min. The collected cells were incubated in PBS containing 1% (*w*/*v*) bovine serum albumin at 25 °C for 1 h [31]. Surface-blocked cells were incubated with 3 mg mL^−^^1^ of goat antibody against the hHGF (R&D, Minneapolis, MN, USA) in PBS for 1.5 h at 25 °C. These cells were then washed with PBS and incubated in 3 mg mL^−^^1^ of AlexaFluor488-conjugated mouse anti-goat IgG antibody (Invitrogen, Waltham, MA, USA) in PBS for 1.5 h at 25 °C, and rewashed.

### 2.4. Immunofluorescence Observation

Yeast cell fluorescence was observed using an Olympus BX51 microscope (Olympus, Tokyo, Japan). In addition, fluorescence units were measured using the SpectraMax M2 Microplate Reader (Molecular Devices, San Jose, CA, USA) with excitation and emission wavelengths of 495 and 519 nm, respectively.

## 3. Results and Discussion

### 3.1. Plasmid Construction and HGF-Displaying Yeast

**Figure 1:**
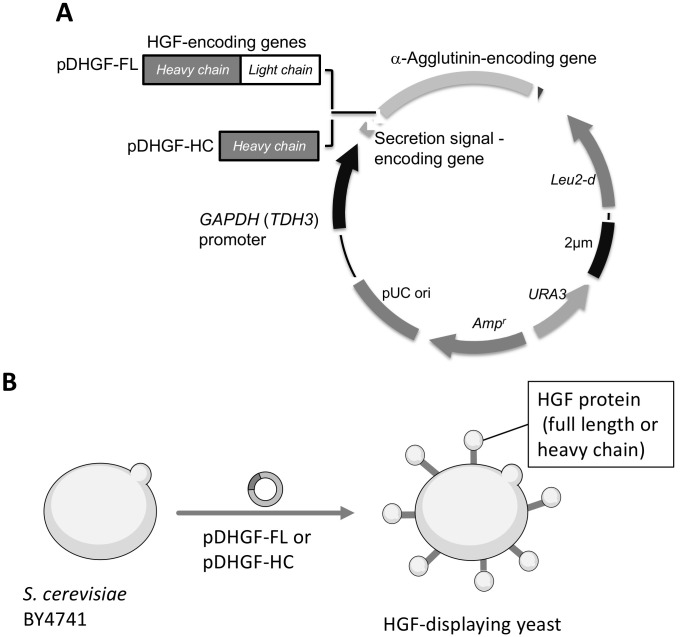
Plasmid constructs for HGF display on the yeast cell surface. (**A**) Genetic fusion of HGF and α-agglutinin. *Leu2-d* in the plasmid is inserted for a multiple copy in the yeast cell. (**B**) Schematics of HGF production on the yeast cell surface. HGF, hepatocyte growth factor.

To display full-length HGF or its heavy chain, we constructed plasmid pDHGF-FL and pDHGF-HC, respectively (Figure 1). Both HGF sequences were confirmed as being correctly cloned into plasmids by comparing them with the GenBank sequences (accession numbers M29145 [32] and L02931 [16]). Next, they were introduced in *S. cerevisiae* BY4741. Confirmation of a successful transformation with these plasmids was performed by auxotrophic selection and colony direct PCR. In the colony PCR, the sizes of fragments corresponding to the full length or heavy chain of HGF were confirmed on the respective plasmids. The strains harboring pDHGF-FL or pDHGF-HC were named BY4741/HGF-FL and BY4741/HGF-HC, respectively.

### 3.2. Immunofluorescence Observation

**Figure 2:**
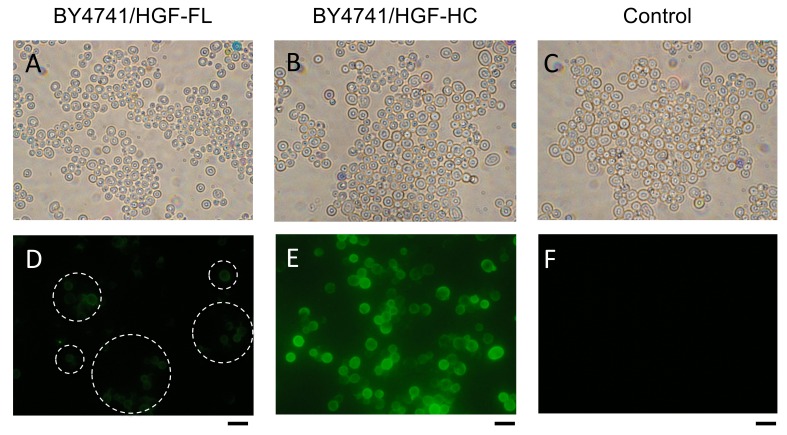
Immunofluorescence microscopic observation of HGF on *Saccharomyces cerevisiae*. (**A**–**C**) Light field micrograph. (**D**–**F**) Fluorescence micrograph. (**A**,**D**) BY4741/HGF-FL; (**B**,**E**), BY4741/HGF-HC; (**C**,**F**), BY4741/pULD1 (control). Dashed circles in (**D**) indicate areas with fluorescent cells. HGF, hepatocyte growth factor. Scale bar = 5 μm.

After the cultivation, the displayed full length and heavy chain of HGF were confirmed by immunofluorescence microscopy (Figure 2). The strain BY4741/HGF-HC had more fluorescence that was emitted by cell surfaces than BY4741/HGF-FL did, suggesting that the heavy chain is more easily displayed on the yeast cell surface than the full-length HGF. 

### 3.3. Cultivation Conditions of HGF-Displaying Yeast

**Figure 3:**
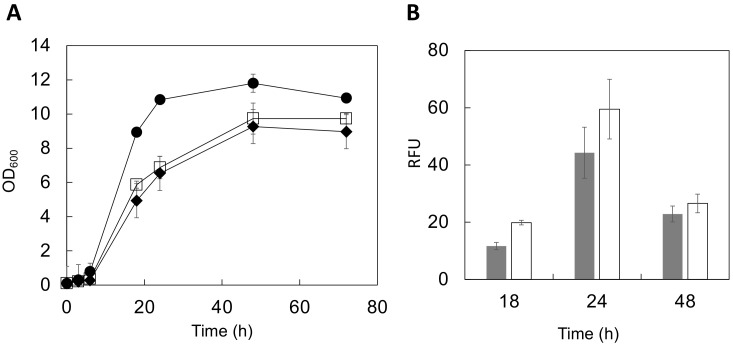
Evaluation of HGF-displaying cells during culture. (**A**) Cell growth. Circle, control; square, BY4741/HGF-FL (full length), diamond, BY4741/HGF-HC (heavy chain). (**B**) The fluorescence intensity from the yeast cell surface was measured and expressed in relative fluorescence units (RFU). Gray, BY4741/HGF-FL; White, BY4741/HGF-HC. Data represent the means ± SD of three independent experiments. SD, standard deviation; HGF, hepatocyte growth factor.

The optical density (OD_600_) was measured up to 72 h after cultivation initiation to evaluate the genetically engineered yeast growth conditions (Figure 3A). During the logarithmic growth phase (6–24 h in culture), cells displaying both full-length HGF and the heavy chain of HGF showed a slow proliferation. In the stationary phase (48–72 h), the growth was 82–89% (full length) or 78–82% (heavy chain) that of the control strain. These data suggest that displaying HGF molecules may affect metabolism associated with yeast cell growth. Additionally, a previous study on the cell surface display of enhanced green fluorescent protein (EGFP) using a similar vector system showed similar results [33]. Considering these cases of cell surface display, the synthesis of HGF’s multiple domains after their translation might consume extra-cellular resources, affecting cell growth.

The fluorescence of yeast cells stained with AlexaFluor488 was analyzed using a multi-well plate reader to observe changes in the relative amounts of HGF displayed on the yeast cell surface during cultivation. Fluorescence intensity is correlated to the amounts of displayed molecules [34,35]. The displayed HGF amounts increased for the first 24 h of the growth cycle (Figure 3B). However, there was a considerable difference between the full-length HGF and the heavy chain of HGF in terms of increasing the displayed molecules. The displayed HGF heavy-chain protein production was approximately 1.4-fold higher than that of the full-length HGF at 24 h of cultivation (Figure 3B). This might be attributed to differences in their molecular sizes. The full-length HGF (84 kDa) is approximately 1.2-fold heavier than the heavy chain (69 kDa), hindering the display of full-length HGF on yeast cells. For a more precise analysis, these comparisons should be done using different anti-HGF IgG antibodies that can recognize different epitopes. Additionally, other yeast strains should also be examined for a high-level display on the yeast cell surface.

## 4. Conclusions

In this study, we demonstrated for the first time that both the full-length and heavy chain of HGF could be displayed on the yeast cell surface. HGF was displayed on the surface depending on the cultivation time for up to 24 h. The fluorescence microscopic observation (Figure 2) and quantitative evaluation using the fluorescence intensity (Figure 3B) consistently suggested that the heavy-chain form is easier to display on the yeast cell surface than the full-length HGF. 

In further research, we will carry out a functional analysis of the administration of HGF-displaying yeast cells to GVHD model animals. The activity of HGF displayed on the yeast cell surface can be optimized since cell surface engineering also provides an easy way to introduce mutations into a displayed protein to produce a library and find variants with enhanced activities [25]. However, further investigations are necessary to develop these engineered yeast cells for oral HGF administration in GVHD model animals. For example, the optimization of yeast culture conditions to increase the amount of protein on the cell surface and the quantification of displayed HGF should be performed. By optimizing these conditions, yeast displaying HGF could be a convenient and inexpensive approach to treating GVHD.
